# Frequency-Dependent Electroformation of Giant Unilamellar Vesicles in 3D and 2D Microelectrode Systems

**DOI:** 10.3390/mi8010024

**Published:** 2017-01-16

**Authors:** Qiong Wang, Xiaoling Zhang, Ting Fan, Zhong Yang, Xi Chen, Zhenyu Wang, Jie Xu, Yuanyi Li, Ning Hu, Jun Yang

**Affiliations:** 1 Key Laboratory of Biorheological Science and Technology (Chongqing University), Ministry of Education, Bioengineering College, Chongqing University, Chongqing 400030, China; wangqiong@cqu.edu.cn (Q.W.); 20126461@cqu.edu.cn (T.F.); 20121913019t@cqu.edu.cn (X.C.); 2 Chongqing Engineering Research Center of Medical Electronics Technology (Chongqing University), Bioengineering College, Chongqing University, Chongqing 400030, China; 3 Department of Laboratory Medicine, Southwest Hospital, Third Military Medical University, Chongqing 400038, China; zyang@tmmu.edu.cn; 4 College of Biomedical Engineering, Chongqing Medical University, Chongqing 400016, China; wangzhenyu20090306@gmail.com; 5 Chongqing Jinshan Science & Technology (Group) Co., Ltd., Chongqing 401120, China; kyxuj@jinshangroup.com (J.X.); liyy@jinshangroup.com (Y.L.)

**Keywords:** electroformation, yield, monodispersity, lipid film

## Abstract

A giant unilamellar vesicle (GUV), with similar properties to cellular membrane, has been widely studied. Electroformation with its simplicity and accessibility has become the most common method for GUV production. In this work, GUV electroformation in devices with traditional 3D and new 2D electrode structures were studied with respect to the applied electric field. An optimal frequency (10 kHz in the 3D and 1 kHz in the 2D systems) was found in each system. A positive correlation was found between GUV formation and applied voltage in the 3D electrode system from 1 to 10 V. In the 2D electrode system, the yield of the generated GUV increased first but decreased later as voltage increased. These phenomena were further confirmed by numerically calculating the load that the lipid film experienced from the generated electroosmotic flow (EOF). The discrepancy between the experimental and numerical results of the 3D electrode system may be because the parameters that were adopted in the simulations are quite different from those of the lipid film in experiments. The lipid film was not involved in the simulation of the 2D system, and the numerical results matched well with the experiments.

## 1. Introduction

Giant unilamellar vesicles (GUVs), a particular type of lipid vesicles, have been widely accepted as cellular membrane models because of their similar properties [1]. The last few decades have witnessed many methods being developed to prepare GUVs. Hydration, the original and simple method, is a completely spontaneous process because of natural flows resulting in very low formation efficiency. Gradually, many other methods were realized that introduced some external energy sources to accelerate the formation process and improve the formation efficiency significantly with the help of a faster convective flow. This kind of method included reverse evaporation [2], sonication [3,4], electroformation [5], extrusion, etc. Electroformation, since first described by Angelova and Dimitrov in 1986, became the most common method for GUV production for its simplicity and accessibility [6]. Different from the hydration method, electroformation involves the presence of an external electric field following the hydration process of a dry lipid film deposited on a substrate.

In electroformation, the most widely used was a sandwiched chamber consisting of two plates of electrodes and a spacer held between them. Based on this, many effective methods have been developed in order to improve the monodispersity and yield of the formed vesicles. For example, microcontact printing based on microfabrication techniques allowed the patterning of controllable-sized lipids and then formed controllable-sized liposomes. Two kinds of microstamps have been used to date: polydimethylsiloxane (PDMS) stamps suitable for organic solvent-soluble lipids [7] and biocompatible hydrogel stamps suitable for aqueous solutions [8]. Analogously, fabricating arrays of microwells on the substrate and trying to achieve the same sized liposomes was another innovative method [9] that can be combined with some techniques such as the electrospray deposition (ESD) method to realize selective patterning of the lipid film. In addition, a coplanar interdigitated electrode system, often used in cell electromanipulation [10,11], micromixers, and micropumps [12,13], has also been used in electroformation [1,14]. Arrays of interdigitated electrodes on the substrate reduced the electrode interval to several tens of micrometers, and thus reduced the required voltage significantly. The last one (the coplanar interdigitated electrode system) appeared to obtain the best yield at present.

For different electroformation devices, the applied electric field also a critical factor is often overlooked. Some research simply mentioned that the frequency exceeding a few hundred Hertz would fail with electroformation and was generally fixed at 10 Hz [5,9]. As a matter of fact, a frequency of thousands of Hertz obtained a better result but a theoretical understanding remains unknown [1,15]. In this work, a traditional method of a 3D electrode system containing a top and bottom electrode and a spacer between them, and the most effective method of a 2D coplanar electrode system which was fabricated with many microelectrode arrays on the substrate were chosen to study experimentally with respect to the applied electric field. An optimal frequency was found in each system. In addition, positive correlation was found between GUV formation and the applied voltage in the 3D electrode system from 1 to 10 V, but was not found in the 2D electrode system. GUVs with a wide size distribution were formed in the 3D electrode system and was improved in the 2D electrode system in both yield and size distribution, which is consistent with those previous results. The experimental results were further confirmed by numerical simulation for the first time in which an optimal frequency was also found in each system.

## 2. Experimental

### 2.1. Materials

l-α-phosphatidylcholine (PC, 1,2-diacyl-sn-glycero-3-phosphocholine) and fluorescent dye (DiI, 1,1′-dihexadecyl-3,3,3′,3′-tetramethylindocarbo-cyanie perchlorate, ex/em: 549/564 nm, Molecular Probes) were purchased from Sigma-Aldrich (St. Louis, MO, USA). Glass slides, polydimethylsiloxane (PDMS) and polymethyl methacrylate (PMMA) were purchased from Kaivo (Zhuhai, China), Dow Corning (Midland, MI, USA), and Yikang (Shanghai, China), respectively. Sucrose (AR) was purchased from Sinopharm Chemical Reagent Co., Ltd. (Shanghai, China). Millipore Milli-Q water with a resistance of 18.25 MΩ·cm at 25 °C was used for solution preparation. All experiments were performed at 25 °C.

### 2.2. 3D Electrode System

**Figure 1:**
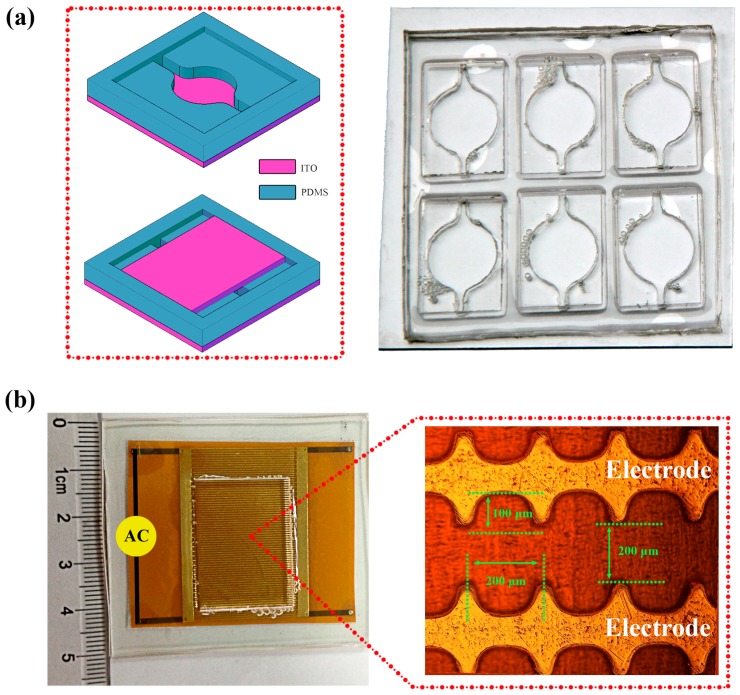
Illustration of the 3D and 2D experimental setup. (**a**) The 3D electrode system consists of a top and a bottom indium tin oxide (ITO) electrode separated by a polydimethylsiloxane (PDMS) spacer. The chip used in experiments contains six subcells and allows several groups of experiments to be carried out at the same time. (**b**) The 2D electrode system with many microelectrode arrays.

The 3D electrode system was designed and constructed as schematically depicted in Figure 1a. It consisted of two indium tin oxide (ITO) planar electrodes (the red parts) and a sandwiched PDMS spacer (the blue part). The reactor consisted of a mixing chamber of 10 mm in diameter and 1 mm in height, and two channels of 2 mm in width. A curve side wall (2 mm radius and 65° angle) was used to connect the mixing chamber and the channels in order to minimize the disturbance of the flow on the lipid film when loading the aqueous solution. The inlet and outlet were left open to avoid bubble formation when the experimental setup was completed. The miniaturized reactor was fabricated based on a well-established soft lithography technique following reference [16]. A PMMA mold was made first by a laser marking machine. A liquid mixture with PDMS and curing agent at a mass ratio of 10:1 was then poured into the PMMA mold, which was degassed to remove the air bubbles and cured at 70 °C for 2 h. The PDMS spacer was thus obtained by separating it from the PMMA mold. Finally, the PDMS spacer and a glass slide (10 cm × 10 cm) were treated with oxygen plasma for 30 s, and then were pressed together. The top glass slides were placed onto the PDMS spacer after lipid film formation to complete the preparation chip.

### 2.3. 2D Electrode System

A microchip with coplanar electrode arrays of symmetrical comb teeth shown in Figure 1b was designed and fabricated based on the flexible printed circuit board (FPCB) technique following reference [17]. The raised comb teeth in the polyimide substrate-based microchip formed a subcell for GUV formation on one hand, and strengthened the electric field on the other hand. The depth of the microchannel was fixed at 35 μm and other dimensions were listed in the diagram. The microelectrode arrays were fabricated on polyimide substrates which was glued on a glass slide. A PDMS spacer was fixed on the microelectrode arrays through curing some PDMS between them. Finally, a general coverslip was placed on the top of the PDMS spacer to hold aqueous solution.

### 2.4. Experimental Procedure

Solution of lipids at a concentration of 4 mg/mL composed of PC and DiI at a 99.5:0.5 mass ratio was prepared in diethyl ether. With the mixed organic solution, 20 μL was dripped onto the substrates of both the 3D and 2D electrode systems shown in Figure S1. The chips after film formation were placed in vacuum overnight to remove the solvent completely followed by covering the upper glasses. Finally, all the chambers were gently filled with 200 mm of sucrose solution and a sinusoidal alternating current (AC) signal was applied to induce vesicle formation.

## 3. Experimental Results

In these electroformation systems, the applied voltage decayed with increasing frequency since the bulk electrolyte was not a pure resistor but a load of resistance and capacitance. So the applied signal was always corrected by using an oscilloscope in each experiment.

### 3.1. GUV Electroformation in the 3D Electrode System

**Figure 2:**
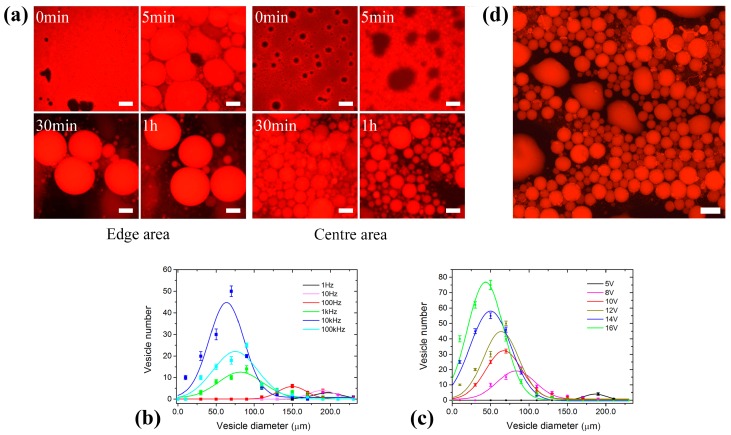
(**a**) The GUV formation process in the edge and central area, respectively, under the electric field of 12 V and 1 kHz. The deposited lipid film swelled into membrane domains first, then closed gradually and detached finally to form vesicles. (**b**,**c**) The scatter diagram and Gaussian fitting curves for GUV diameters at different frequencies (12 V) and voltages (10 kHz), respectively. Where, the GUV number was obtained by specifying a box (1 mm × 1 mm) at the center and edge, respectively, and then added them up. The GUVs with diameter within the interval of (0, 20) were classified into *d* = 10 μm, and so on. (**d**) The formed GUVs at 16 V and 10 kHz. The scale bar is 100 μm.

First, the amplitude was fixed at 12 V with varying frequency from 1 Hz to 10^5^ Hz (×10 for the interval) to study the effect of the applied electric field on the GUV electroformation. At 10 Hz, an undesired result was obtained with sporadic super giant vesicles at the edge area and only swelling lipid membranes in the center. A better effect was observed as the frequency increased (Figure 2a, the GUV formation process in the edge and central area under 12 V and 1 kHz were displayed). Vesicles with a wide size distribution were generated, and big ones distributed at the edge area while small ones distributed in the center. This may be due to the non-uniform thickness of the lipid film in some degree. Both the monodispersity and the yield of the generated vesicles were continuously increased with increasing frequency until 10 kHz but did not change too much when the frequency continued to increase (Figure 2b). The experiment for each given frequency were carried out at least three times.

The frequency was then fixed at 10 kHz to study the effect of the applied voltage (Figure 2c). No vesicles except some swelling lipid membranes were formed in the center when the voltage was less than 5 V. More and more GUVs began to generate with increasing electric field strength and decreased in the average size when the amplitude was varied from 5 V to 16 V (Figure 2d, 16 V, 10 kHz).

### 3.2. GUV Electroformation in the 2D Electrode System

**Figure 3:**
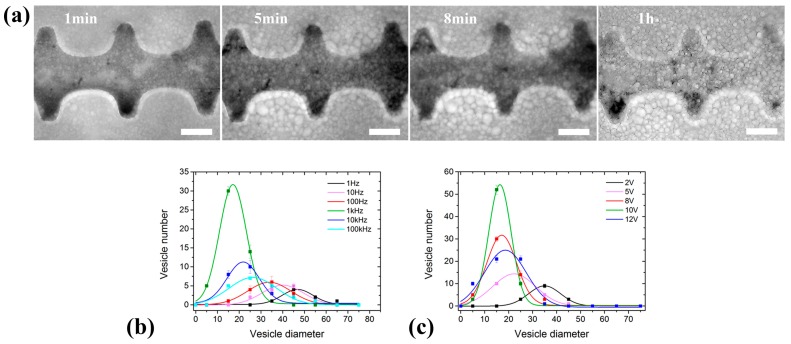
(**a**) The GUV formation process in the 2D electrode system at 8 V and 1 kHz. (**b**,**c**) The scatter diagram and Gaussian fitting curves for GUV diameters at different frequencies (8 V) and voltages (1 kHz), respectively. Where, the vesicle number was obtained in a subcell of the microchip. The GUVs with diameter within the interval of (0, 10) were classified into *d* = 5 μm, and so on. The scale bar is 100 μm.

Preparation efficiency was improved significantly in the 2D electrode system (Figure 3a). When the amplitude was fixed at 8 V, the yield and the monodispersity of the formed GUVs increased with increasing frequency, and were maximized at 1 kHz (Figure 3b). When the frequency exceeded 1 kHz, both the yield and monodispersity of the formed GUVs decreased significantly. Compared with that in the 3D electrode system, the yield of the generated GUVs was obviously improved (Figure 2d and Figure 3a). This may be due to the narrowed electrode interval in the 2D electrode system strengthening the electric field. The frequency was subsequently fixed at 1 kHz to study the effect of the applied voltage. It should be noted that the yield and the monodispersity of the formed vesicles increased when varying amplitude from 1 V to 10 V (Figure 3c). However, more and more lipid debris was observed gradually when the amplitude exceeds 10 V, which however has not been observed in the 3D electrode system.

In addition to the external electric field, the monodispersity of the generated GUVs in the 2D system is also related to the configuration of the microchip, such as the width of the electrode and the electrode interval. Therefore, a further improvement of the monodispersity of the generated GUVs could be achieved by optimizing the design of the microchip.

## 4. Numerical Analysis

Lipids deposited on the substrate rearrange to form regular bilayer sheets which would continuously fuse without external disturbance. The larger the external force, the earlier the inter-membrane fusion would be stopped, and the smaller and more GUVs would be formed. This phenomenon was captured by us and presented in another article. AC Electroosmotic flow (ACEO) of the aqueous solution, which is formed by the migration of net charges in the diffuse layer under a tangential electric field [11,18,19,20], was widely accepted as the main driving force for electroformation [21], and often calculated by the Helmholtz-Smoluchowski (HS) velocity,

(1) $$u=-\frac{\varepsilon_{0}\varepsilon_{r}\zeta }{\mu }E$$

where, ε_0_ is the dielectric constant of the vacuum (8.85 × 10^−12^ F/m) and ε*_r_*, μ are the relative dielectric constant and viscosity of the solution, respectively. **E** and ζ are the electric field vector and zeta potential, respectively. Two models in this work were built for calculating the EOF in the preparation chamber. The load the lipid film experienced from the ACEO was calculated by

(2) $$T_{s}=-n\cdot \left(-pI+\mu \left(\nabla u\right)\right)$$

which was a sum of pressure and viscous forces and was used to characterize the effect of the applied electric field on GUV electroformation. The larger this load, the larger the yield of the generated GUVs.

**Figure 4:**
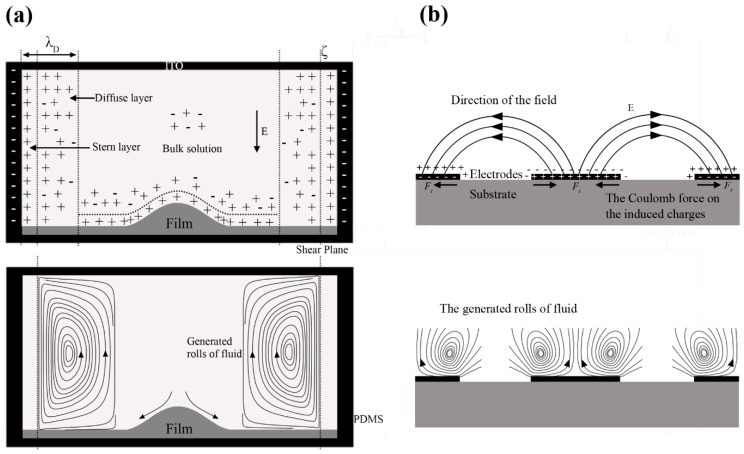
The formation mechanism of the ACEO in the 3D (**a**) and 2D (**b**) electrode system.

In the 3D electrode system, the functional electric double layer (EDL) was formed near the chamber wall (made of PDMS) ignoring the deformation of the lipid film shown as Figure 4a. The formed EOF under an AC field thus depended on the zeta potential (ζ) of the chamber wall which was usually considered as uniform and constant under some assumptions [22] and was set to 50 × 10^−3^ V in this work. In the 2D electrode system, the AC applied field might act on its own induced diffuse charges near a polarizable electrode (Figure 4b). Compared with the 3D electrode system, the 2D one could concentrate the electric field on the substrate and the functional zeta potential was much higher related to the applied voltage.

**Figure 5:**
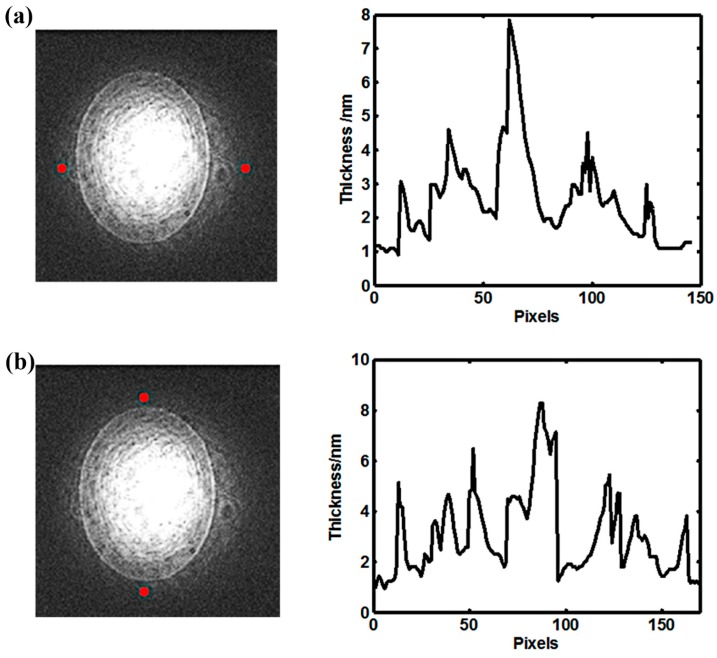
The thickness distribution of the lipid film deposited on the substrate including the transverse distribution (**a**) and the vertical distribution (**b**).

The conductivity of the used aqueous solution (200 mm sucrose solution) was measured by using the conductivity detector (METTLER TOLEDO, Columbus, OH, USA) which was 5 × 10^−4^ S/m. The properties of the liquid were assumed to be constant because the Joule-heating produced by the applied electric field can be neglected when the conductivity was so low. The properties of the aqueous solution, such as the relative dielectric constant of the solution ε*_r_* ~ 80, density ρ ~ 1000 kg/m^3^, viscosity μ ~ 9 × 10^−4^ Pa∙s, temperature *T* ~ 293.15 K, and the dielectric constant of the lipid film ε*_m_* ~ 5, were adopted and assumed to be constant. The thickness of lipid film was measured by a homemade surface plasmon resonance (SPR) imaging device [23]. During the detection, the organic solution of the lipid mixture was diluted 1000 times to ensure the film thickness within the effective detection range of the device. The results were shown in Figure 5 and the average thickness of the lipid film *d_m_* was 2 μm and assumed to be flat in the numerical simulation.

In addition, it should be noted that the lipid film covered the whole substrate of the 3D electrode system (Figure S1a). The complex impedances of the lipid film is *Z_m_* = 1/(*j*ω*C_m_*), *C_m_* = ε_0_ε*_m_*/*d_m_* is the capacitance of the lipid film. The complex impedances of the EDL are *Z_D_* = 1/(*j*ω*C_D_*) and *C_D_* = ε_0_ε*_r_*/*d_D_* and *d_D_* is the capacitance and the thickness of the EDL near the electrode surfaces, respectively. Together with *d_m_*/*d_D_* ~ 10^3^ and ε*_m_*/ε*_r_* ~ 10, the impedance of the lipid film is thus several orders of magnitude (>10^2^) larger than that of the EDL. Considering the lipid film and EDL are two capacitors and in series in the 3D electrode system, the EDL can thus be neglected. Whereas, in the 2D electrode system, the lipid film only deposited in the electrode intervals (Figure S1b) and did not affect the electric circuit thus could be neglected. Overall, the model of the 3D electrode system consists of a resistance (the bulk solution) and a capacitor (the lipid film) in series, and so does the model of the 2D one (the bulk solution and the EDL). The lipid film in the 3D system, because of its infinitesimal thickness compared with the bulk solution, an effective boundary condition was thus necessary to avoid failure on meshing, as well as the EDL in the 2D electrode system. Laminar flow was adopted in both models because the Reynolds number is extremely low [12,24]. All the calculations were carried out in a commercial finite element package, COMSOL Multiphysics 4.4 (COMSOL Inc., Los Angeles, CA, USA).

### 4.1. Modeling of the Systems

#### 4.1.1. The 3D Electrode System

**Figure 6:**
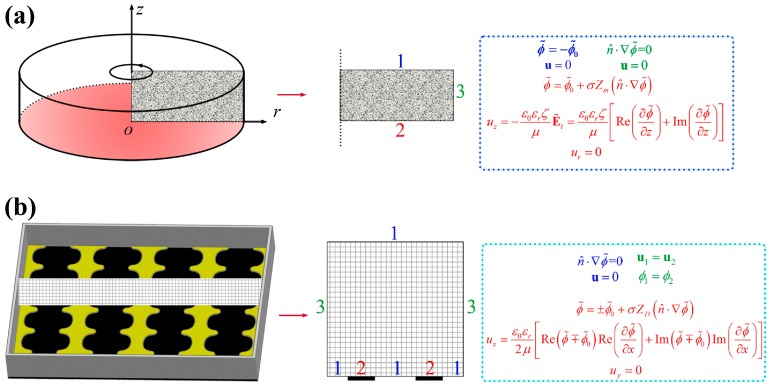
The schematic diagrams of the 3D (**a**) and 2D (**b**) electrode system, and their corresponding boundary conditions.

The cylindrical cell in the 3D electrode system, because of its characteristic of symmetry, was simplified into a 2D symmetric model. Only the shaded area was calculated and all the results could be obtained by rotating this plane about *z*-axis (Figure 6a). On the bottom electrode, the film and the electrode were combined into a boundary, and a Robin-type boundary condition which involved the conductivity, permittivity, and thickness of the film was used. The conductivity of the aqueous solution (5 × 10^−4^ S/m) was larger than that of the lipid film (~10^−6^ S/m [25], and ~5 × 10^−7^ [26,27]) by several orders of magnitude. The conductivity property of the lipid film was thus neglected during the computation.

Equations in the frequency domain in *Electric Currents* were used to obtain the steady sinusoidal response to the external AC signal [28,29,30]. The governing equation for the electric field in the frequency domain can be expressed as:

(3) $$\nabla \cdot \left(\sigma +j\omega \varepsilon \right)\nabla \tilde{\phi }=0$$

where $\tilde{\phi }$ was the phasor of electric potential which was a complex with ϕ=Re(ϕ˜ejωt), and ω = 2π*f* was the angular frequency of the AC signal. When neglecting the dielectric properties of the solution, Equation (1) can be simplified as:

(4) $$\nabla^{2}\tilde{\phi }=0$$

As the lipid film was deposited on the bottom surface, the Robin-type boundary condition $\tilde{\phi }$ was imposed on the bottom electrode surface,

(5) $$\frac{\tilde{\phi }-{\tilde{\phi }}_{0}}{Z_{m}}=\sigma \left(\hat{n}\cdot \nabla \tilde{\phi }\right)$$

therefore,

(6) $$\tilde{\phi }={\tilde{\phi }}_{0}+\sigma Z_{m}\left(\hat{n}\cdot \nabla \tilde{\phi }\right)$$

and no lipid film was on the top surface, then the boundary condition on the top electrode was set to $\tilde{\phi }=-{\tilde{\phi }}_{0}$. The PDMS wall was considered as insulated, $\hat{n}\cdot \nabla \tilde{\phi }=0$, shown in Figure 6a. Here, ${\tilde{\phi }}_{0}$ was the amplitude of the applied AC signal with ϕ0=Re(ϕ˜0ejωt), and $\hat{n}$ was the unit vector normal to the membrane surface.

Due to extremely low Reynolds number for the flow, the Stokes equation was used to describe the flow field:

(7) ∇⋅u=0

(8) $$-\nabla p+\mu \nabla^{2}u=0$$

with a boundary condition of the HS slip velocity on the PDMS wall [31],

(9) uz=−ε0εrζμE˜t=ε0εrζμ[Re(∂ϕ˜∂z)+Im(∂ϕ˜∂z)]

(10) $$u_{r}=0$$

and no slip on the two electrode surfaces. Here *u* was the time-averaged velocity field with *z* component *u_z_*, and *r* component *u_r_*. *p* was the pressure, and ${\tilde{E}}_{t}$ was the *z* component of the electric field vector near the PDMS wall.

#### 4.1.2. The 2D Electrode System

The length of the microelectrode was infinite compared with the electrode intervals within the 2D electrode system, which was thus simplified as a vertically 2D model as that in Figure 6b. Average values of the electrode width (200 μm) and interval (300 μm) were used. ACEO flow near the electrode surface was formed and may be much stronger than the EOF formed in the 3D electrode system because of a larger zeta potential. Similarly to the 3D electrode system, a Robin-type boundary condition was also applied to the EDL here.

Equations in the frequency domain were also used here similar to that of the 3D electrode system, with the Robin-type boundary condition $\tilde{\phi }$ at the electrode surfaces,

(11) $$\tilde{\phi }=\pm {\tilde{\phi }}_{0}+\sigma Z_{D}\left(\hat{n}\cdot \nabla \tilde{\phi }\right)$$

as well as periodic condition and electric insulation on the vertical boundaries and the remaining boundaries, respectively. Boundary conditions for the flow field included a HS slip velocity parallel to the electrode surfaces,

(12) ux=ε0εr2μ[Re(ϕ˜∓ϕ˜0)Re(∂ϕ˜∂x)+Im(ϕ˜∓ϕ˜0)Im(∂ϕ˜∂x)]

(13) $$u_{y}=0$$

and periodic flow condition and no slip on the vertical boundaries and the remaining boundaries, respectively.

### 4.2. Simulation Results and Discussion

#### 4.2.1. The 3D Electrode System

**Figure 7:**
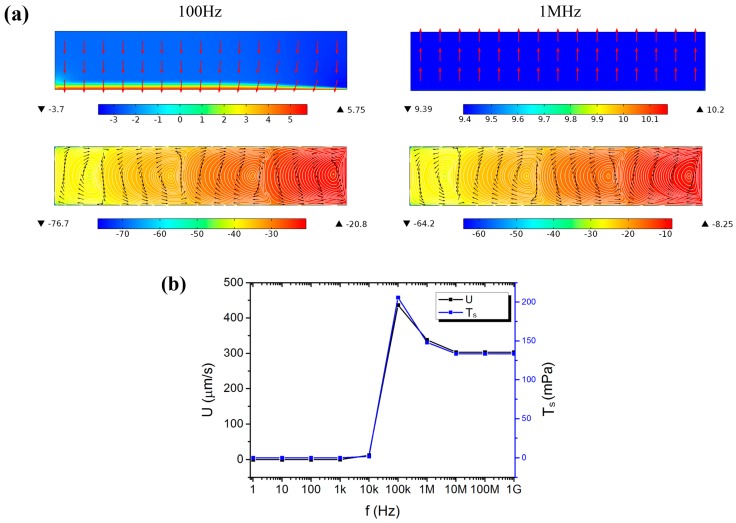
Numerical results of the 3D electrode system. (**a**) The electric field and the formed EOF in a vertical section at 100 Hz and 1 MHz, respectively. The surface plot used a logarithmic scale. (**b**) The frequency-dependent maximum value of the velocity amplitude (*U*) and the load (T_s_) the lipid film experienced from the ACEO.

The impact of frequency (from 1 Hz to 1 GHz, ×10 for interval) was calculated when ϕ_0_ was fixed at 12 V. Vortexes were formed in the preparation chamber. Most of the applied voltage dropped across the lipid film and the bulk electrolyte when the frequency was lower and higher than 100 kHz, respectively. When the frequency was lower than 100 kHz, the electric field in the bulk electrolyte was reversed with respect to the applied electric field, as well as the formed vortex shown in Figure 7a. This may be due to the lipid film being treated as a capacitor in this work which would store the energy and discharge to the solution at low frequency. It implies that the reversal of the direction of the formed ACEO can be induced by the frequency. The maximum value of the load (T_s_) the lipid film experienced from the ACEO was shown in Figure 7b. It maximized at 100 kHz, which suggested that a characteristic frequency did exist in the 3D electrode system. In addition, the ACEO also maximized at this frequency, which was not consistent with the previous result that ACEO predominated at low frequencies and attenuated with increasing frequency [22].

However, the characteristic frequency of 100 kHz was different from the experimental result where the optimal frequency was 10 kHz. This may be due to the present model adopting the properties of cell membrane, while the lipid film in experiments was not regular multiple bilayers but lipid aggregates with many interspaces. The aggregate interspaces would allow electrolyte to penetrate through and lead to a large discrepancy in the electrical properties (conductivity and dielectric constant) from the cell membrane. In addition, when the frequency exceeded the characteristic one (100 kHz), T_s_ slightly decreased and in some degree confirmed the experimental result that the yield of the formed GUVs did not decrease that much when the frequency exceeded 10 kHz. The characteristic frequency was expressed previously as [28]:

(14) $$\omega_{0}\approx \frac{\sigma }{\varepsilon_{0}\varepsilon_{m}}\frac{d_{m}}{h}$$

where *h* is the electrode gap.

#### 4.2.2. The 2D Electrode System

**Figure 8:**
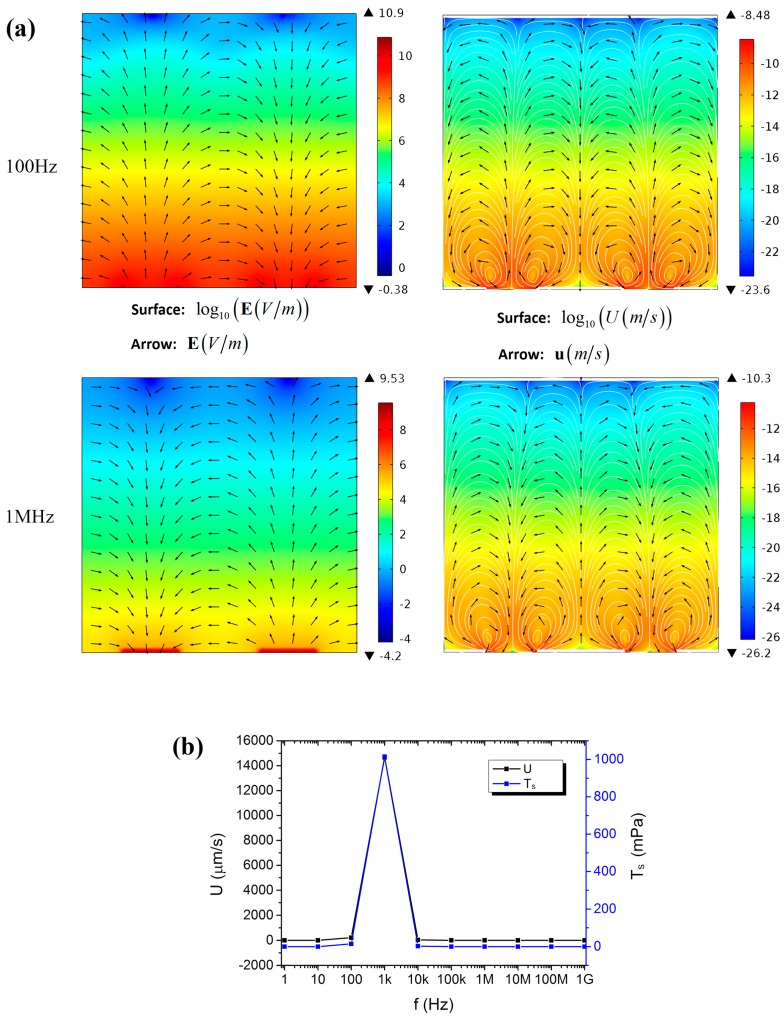
Simulation results of the 2D electrode system. (**a**) The electric field and the formed EOF in a vertical section at 100 Hz and 1 MHz, respectively. The surface plot used a logarithmic scale. (**b**) The frequency-dependent maximum value of the velocity amplitude (*U*) and the load (T_s_) the lipid film experienced from the ACEO.

The impact of frequency (from 1 Hz to 1 GHz, ×10 for interval) was also calculated in the 2D electrode system when ϕ_0_ was fixed at 3.6 V in order to obtain the same external electric field as that in the 3D one (1.2 × 10^4^ V/m). Two symmetric counter-rotating rolls of fluid flow were formed on each electrode surface. Like that of the 3D electrode system, most of the applied voltage dropped across the EDL and the bulk electrolyte when the frequency was lower and higher than 1 kHz, respectively. When the frequency was lower than 1 kHz, the electric field in the bulk electrolyte was reversed with respect to the applied one, as well as the formed ACEO (Figure 8a). The maximum value of the load the lipid film experienced (T_s_) was shown in Figure 8b and maximized at 1 kHz. This suggested that a characteristic frequency also did exist in the 2D electrode system. When the frequency exceeded the characteristic one (1 kHz), T_s_ decreased sharply and in some degree confirmed the experimental result that the yield of the generated GUVs decreased very remarkably when the frequency exceeded 1 kHz.

It should also be noted that the simulation results of the 3D and 2D electrode systems were different even though the same external electric field was applied. At the characteristic frequency, the load the lipid film experienced in the 2D electrode system was much higher than that in the 3D one. Therefore, more GUVs were formed in the 2D electrode system than in the 3D one, and a saturation phenomenon was observed in the 2D but not in the 3D electrode system experimentally. As mentioned above, this may be also due to the strengthened electric field by the narrowed electrode interval in the 2D electrode system.

## 5. Conclusions

Traditional 3D and a 2D electrode systems were investigated experimentally with respect to the applied electric field on electroformation of GUVs. An optimal frequency was found in each system (10 kHz in 3D system and 1 kHz in 2D system) under which a good yield and monodispersity of the generated vesicles can be achieved. This was not consistent with those reported results that low frequency was more suitable for GUV electroformation. The results were confirmed further by numerical modeling in which a characteristic frequency did exist for the resistor–capacitor (RC) circuit in each system (100 kHz in 3D system and 1 kHz in 2D system). Under this frequency, the load the lipid film experienced from the formed ACEO can be maximized. The discrepancy between the experimental (10 kHz) and numerical (100 kHz) results in the 3D electrode system were attributed to the uncertainty of the lipid film properties, since the lipid film in GUV electroformation was greatly different from the cell membrane. The 2D electrode system did not involve the lipid film and thus matched with the experiments better.

## Supplementary Materials

The following are available online at www.mdpi.com/2072-666X/8/1/24/s1, Figure S1: Lipid film deposited on the substrate of the 3D and 2D electrode system, respectively.
