# Supplementary material for: Frequency-Dependent Electroformation of Giant Unilamellar Vesicles in 3D and 2D Microelectrode Systems
Source: Micromachines (Basel). 2017 Jan 16;8(1):24. doi: 10.3390/mi8010024 (PMC6190065; doi:10.3390/mi8010024)
Supplement: Supplementary file 1 [file micromachines-08-00024-s001.pdf]

Supplementary Materials: Frequency-Dependent Electroformation of Giant Unilamellar Vesicles in 3D and 2D Microelectrode Systems

Qiong Wang, Xiaoling Zhang, Ting Fan, Zhong Yang, Xi Chen, Zhenyu Wang, Jie Xu, Yuanyi Li, Ning Hu and Jun Yang

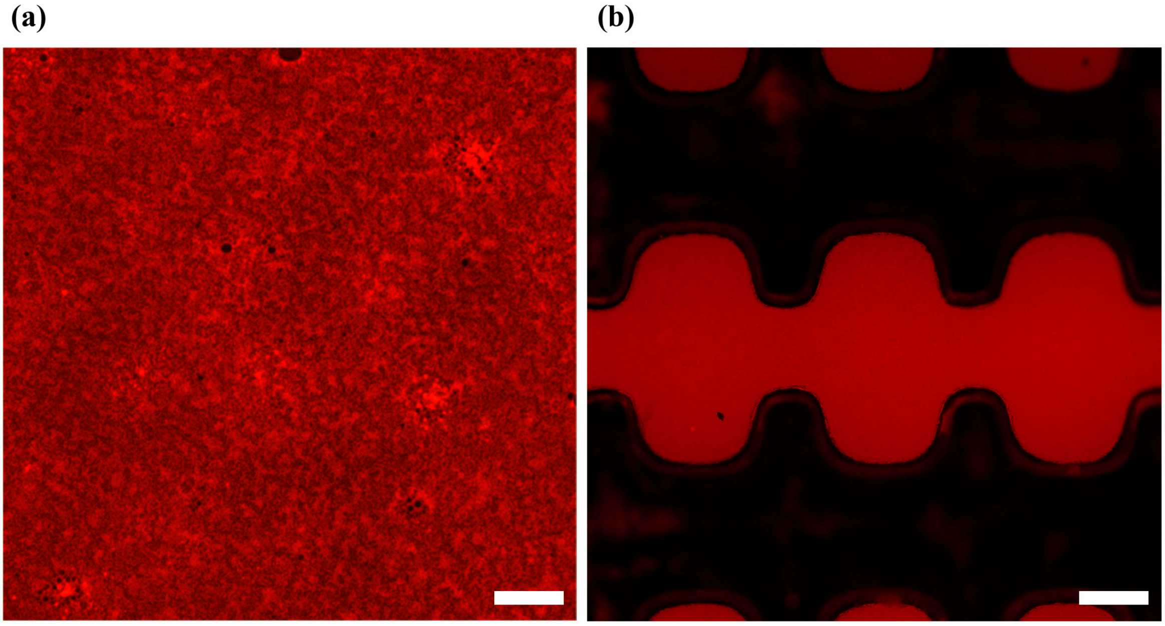

Figure S1. Lipid film formed in the 3D (**a**) and 2D (**b**) microelectrode system.
